# Kup-mediated Cs^+^ uptake and Kdp-driven K^+^ uptake coordinate to promote cell growth during excess Cs^+^ conditions in *Escherichia coli*

**DOI:** 10.1038/s41598-017-02164-7

**Published:** 2017-05-18

**Authors:** Ellen Tanudjaja, Naomi Hoshi, Yi-Hsin Su, Shin Hamamoto, Nobuyuki Uozumi

**Affiliations:** Department of Biomolecular Engineering, Graduate School of Engineering, Tohoku University, Aobayama 6-6-07, Sendai, 980-8579 Japan

## Abstract

The physiological effects of caesium (Cs) on living cells are poorly understood. Here, we examined the physiological role of Cs^+^ on the activity of the potassium transporters in *E*. *coli*. In the absence of potassium (K^+^), Kup-mediated Cs^+^ uptake partially supported cell growth, however, at a much lower rate than with sufficient K^+^. In K^+^-limited medium (0.1 mM), the presence of Cs^+^ (up to 25 mM) in the medium enhanced growth as much as control medium containing 1 mM K^+^. This effect depended on the maintenance of basal levels of intracellular K^+^ by other K^+^ uptake transporters. Higher amounts of K^+^ (1 mM) in the medium eliminated the positive effect of Cs^+^ on growth, and revealed the inhibitory effect of high Cs^+^ on the growth of wild-type *E*. *coli*. Cells lacking Kdp, TrkG and TrkH but expressing Kup grew less well when Cs^+^ was increased in the medium. A *kdp* mutant contained an increased ratio of Cs^+^/K^+^ in the presence of high Cs^+^ in the medium and consequently was strongly inhibited in growth. Taken together, under excess Cs^+^ conditions Kup-mediated Cs^+^ influx sustains cell growth, which is supported by intracellular K^+^ supplied by Kdp.

## Introduction

Caesium (Cs) is an alkali metal in group 1 of the periodic table that is not an essential element for most living cells. Cs has gained special world-wide attentions due to the release of considerably large amounts of radioactive caesium (^134^Cs and ^137^Cs) after the power plant accidents in Chernobyl in 1986 and Fukushima in 2011. This has led to studies of the environmental impacts of Cs^+^ contamination^1, 2^, decontamination attempts^3^ and bioremediation efforts including the search for microorganisms highly tolerant to Cs^+^^4, 5^. The physiological effect on living organisms is poorly understood^6^, but toxicity of Cs^+^ to cells has been reported in microorganisms, animal cells and plant cells^5, 7, 8^. Application of Cs^+^ increased the doubling time of green algae and cyanobacteria^9, 10^. Due to the similar chemical properties of Cs^+^ and K^+^, it has been proposed that Cs^+^ enters the cell via K^+^ transport systems^6, 11^. However, Cs^+^ is frequently used as a K^+^ channel blocker, and most K^+^ transport systems do not facilitate the uptake of Cs^+^ instead of K^+^^12, 13^.

K^+^ is the most abundant cation in prokaryotic and eukaryotic cells. K^+^ plays an important role in the maintenance of intracellular osmolality, formation of membrane potential and regulation of enzyme activity^14^. K^+^ uptake transport systems can be divided into four classes; K^+^ channels, Trk/Ktr/HKT, Kdp and Kup/HAK/KT^15^. With respect to the conserved selectivity filter motif and their membrane topology, Trk and Kdp belong to the same family of K^+^ transporters. The structure of Kup has not been conclusively determined^16^. In *Escherichia coli*, only Kup has been reported to serve as a possible Cs^+^ uptake route but not any of the other major K^+^ uptake systems: Kdp and Trk (TrkG and TrkH)^11^. Kup is a low affinity K^+^ transport system that also transports Rb^+^ and Cs^+^^11^. Kup participates in adaptation to high osmolality stress^17^. Kup was originally isolated in a *trk*D mutant study as a K^+^ uptake system belonging to a different class than TrkG and TrkH^18^. Kup (predicted molecular mass: 69 kDa) consists of an N-terminal transmembrane region (approximately 440 amino acids) and a relatively long (approximately 180 amino acids) C-terminal hydrophilic domain^16, 19^. Several critical residues for Kup/HAK/KT transport function have been identified^16^. On the other hand, the response of *E*. *coli* to Cs^+^ remains to be elucidated. In plants, several groups reported Cs^+^ uptake actvity by members of the Kup/HAK/KT family. Barley HvHAK1 and Arabidopsis AtHAK5 have high affinity for K^+^^20^. AtHAK5, a high affinity type K^+^ transport system, contributes to Cs^+^ influx into roots under K^+^-depleted conditions^20, 21^. The expression of *AtHAK5* is induced 9-fold due to K^+^-starvation in *Arabidopsis thaliana* ^8^. Here, we report that at high concentrations Cs^+^ inhibited growth but that Cs^+^ uptake mediated by Kup could partially substitute for K^+^ inside *E*. *coli* cells under conditions where the K^+^ concentration in the medium was limiting. This positive effect was dependent on K^+^ uptake transporters. An assay with mutant strains revealed that Kdp increased the internal K^+^ content in the cells. Both Kup and Kdp contributed to increased tolerance to both excess Cs^+^ and lack of K^+^ in the medium.

## Results

### Cs^+^ can substitute for K^+^ in supporting *E*. *coli* growth under K^+^-depleted conditions

**Figure 1:**
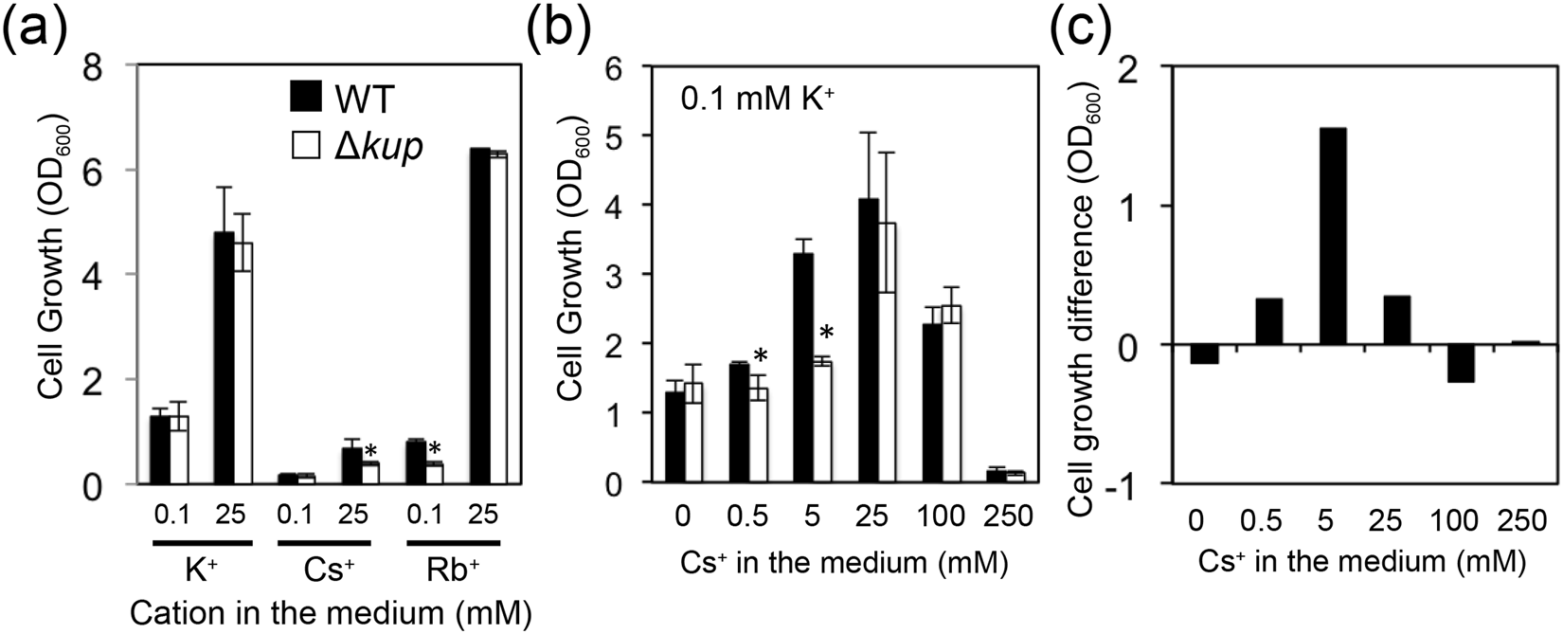
Growth tests of *E*. *coli* WT and Δ*kup* in minimal medium. *E*. *coli* WT (black bars) and Δ*kup* (white bars) grown in minimal medium containing 0.1 mM K^+^ were collected by centrifugation, washed with K^+^-free buffer, then inoculated into minimal medium supplemented with K^+^, Cs^+^ or Rb^+^ (**a**), or with 0.1 mM K^+^ and various concentrations of Cs^+^ (**b**). Cell growth was determined after 24 h-incubation at 30 °C. *Represents significance by Student’s *t*-test comparing WT to Δ*kup* at the same concentration (*P* < 0.05). (**c**) Difference in growth (OD_600_) between WT and Δ*kup* calculated from the data in Fig. 1b.

We tested whether Cs^+^, which is a similar alkali metal element, can act as a substitute for K^+^ during *E*. *coli* growth. In parallel, we tested the effect of Rb^+^ on *E*. *coli* growth since the elements can substitute potassium^22^. Growth tests of *E*. *coli* wild type (WT) and a knockout mutant of *kup* (Δ*kup*) were carried out in minimal medium supplemented with K^+^, Cs^+^ or Rb^+^ (Fig. 1a). In the case of medium supplemented with Cs^+^ a background level of 4.6 μM K^+^ as determined by flame photometry was present. Overall, at the same concentrations, Cs^+^ did not support the same amount of growth of either WT or Δ*kup* as K^+^. However, increasing the amount of Cs^+^ in the medium from 0.1 mM to 25 mM increased growth for both strains (Fig. 1a). The growth increase was higher for the WT (3.8-fold) than for the Δ*kup* strain (2.6-fold). On the other hand, Rb^+^ could take over K^+^ under the conditions (Fig. 1a). These results indicate that Kup contributed to Cs^+^ uptake to sustain growth under depleted-K^+^ conditions.

To further evaluate utilization of Cs^+^ in *E*. *coli* under K^+^-limited condition, we examined the growth of WT and Δ*kup* in minimal medium supplemented with low K^+^ (0.1 mM) and various concentrations of Cs^+^. The result showed that addition of Cs^+^ up to 25 mM Cs^+^ increased the growth of both strains (Fig. 1b). Higher concentrations of Cs^+^ (100–250 mM) impaired the growth. In order to determine the contribution of Kup to the observed growth, we subtracted the OD_600_ of the Δ*kup* strain from that of the WT at each Cs^+^ concentration (Fig. 1c). This illustrated that *E*. *coli* utilized Kup for Cs^+^ uptake at concentrations of 0.5–25 mM effectively under K^+^-limited (0.1 mM) conditions and optimally at 5 mM. Under low K^+^ conditions, Kup therefore contributed to sustaining cell growth against excess external Cs^+^.

### Kup contributes to uptake of Cs^+^ into *E*. *coli* at low external K^+^ concentrations

**Figure 2:**
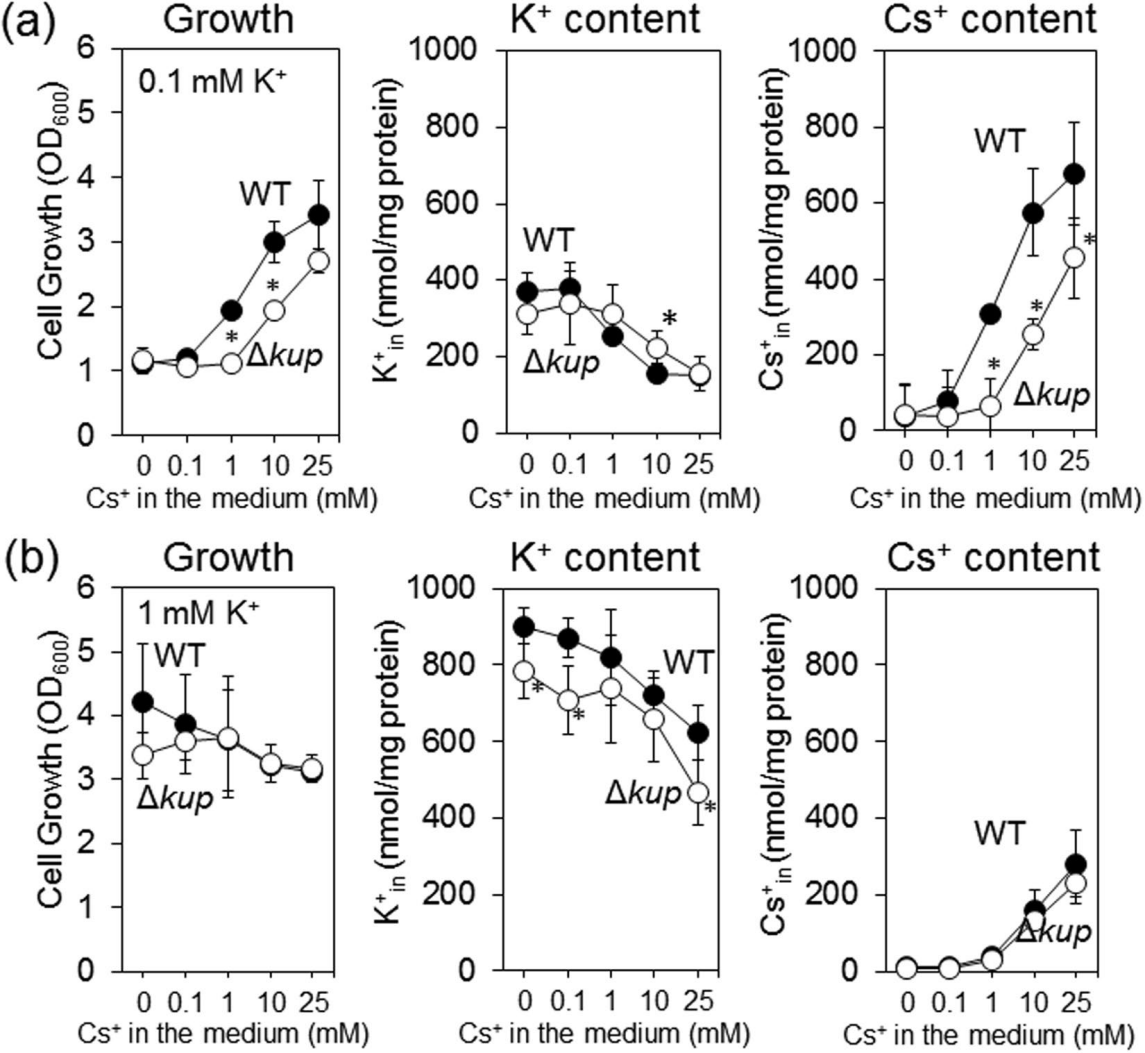
Determination of growth and K^+^ or Cs^+^ accumulation of *E*. *coli* WT and Δ*kup*. *E*. *coli* WT and Δ*kup* were grown in minimal medium supplemented with 0.1 mM K^+^ (**a**) or 1 mM K^+^ (**b**) and various Cs^+^ concentrations for 24 h at 30 °C. At that time, OD_600_ as well as intracellular K^+^ and Cs^+^ content were determined. The error bars represent mean ± S.D. (n = 4). *Represents significance by Student’s *t*-test comparing WT to Δ*kup* at the same concentration (*P* < 0.05).

To further elucidate the role of Kup in taking up Cs^+^ as a substitute for K^+^ in *E*. *coli*, we measured the intracellular amount of K^+^ and Cs^+^ during the growth of *E*. *coli*. In medium with 0.1 mM K^+^ (Fig. 2a), the growth of both WT and Δ*kup* increased at the same time as the Cs^+^ content in the cells in correlation with the Cs^+^ concentration in the medium. In contrast, the intracellular K^+^ was reduced as the amount of Cs^+^ increased. Nevertheless, the K^+^ content remained above 152 nmol/mg-proteins. The other K^+^ uptake transporters, TrkG, TrkH and Kdp, might contribute to K^+^ uptake in K^+^-containing medium. At high K^+^ (1 mM) (Fig. 2b), the positive effect of Cs^+^ on the cell growth of WT and Δ*kup* disappeared and at higher concentrations of Cs^+^ growth of both *E*. *coli* strains was moderately inhibited. Interestingly, the growth of the WT was higher than that of Δ*kup* in medium without Cs^+^ or with 0.1 mM Cs^+^ (Fig. 2b). This suggested that Kup functioned as a K^+^ uptake system. The reduced growth of Δ*kup* in medium with 0 to 25 mM Cs^+^ was correlated with a decrease in intracellular K^+^ (Fig. 2b). This result also illustrated the contribution of other K^+^ uptake transporters to K^+^-loading into the cells. In contrast, the difference in Cs^+^ accumulation between both strains was relatively small, indicating that high K^+^ in the medium prevented Kup-mediated Cs^+^ uptake (Fig. 2b). These results showed that Kup-driven Cs^+^ uptake contributed to cell growth only under K^+^-limited conditions but had an adverse effect on growth under K^+^-sufficient conditions.

### Kup activity impaired growth of *E*. *coli* lacking TrkG, TrkH and Kdp under high external Cs^+^ concentrations

**Figure 3:**
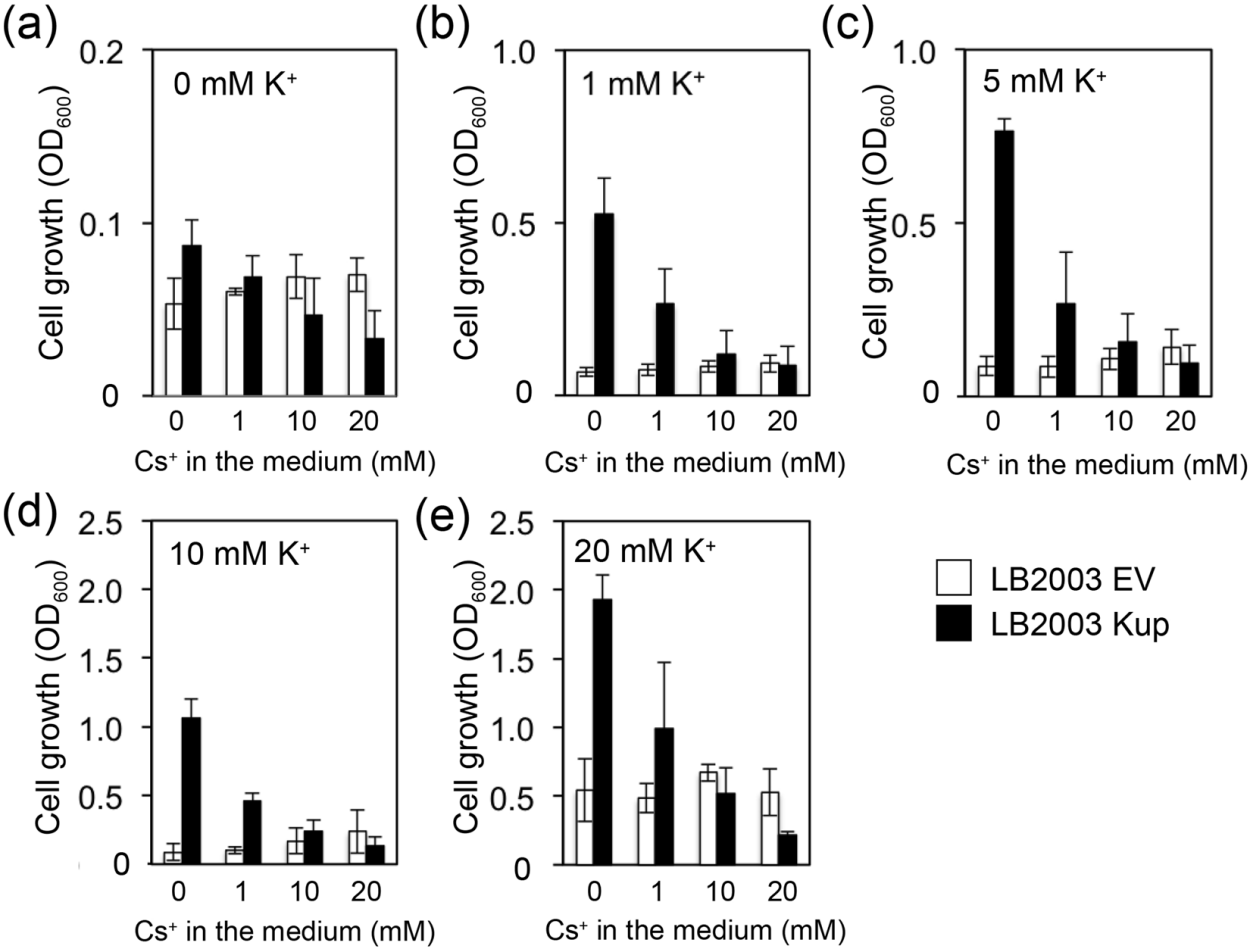
Comparison of growth of the K^+^ uptake system-deficient *E*. *coli* strain LB2003 transformed with Kup or the empty vector (EV) at various concentrations of K^+^ and Cs^+^. Cells grown in minimal medium containing 20 mM K^+^ were collected by centrifugation, washed with K^+^-free buffer and then inoculated into minimal medium supplemented with combinations of various concentrations (0–20 mM) of K^+^ and Cs^+^. Growth was determined after 24 h-incubation at 30 °C. (**a**) 0 mM K^+^, (**b**) 1 mM K^+^, (**c**) 5 mM K^+^, (**d**) 10 mM K^+^, (**e**) 20 mM K^+^. The error bars represent mean ± S.D (n = 3). LB2003 strain containing Kup (black bars) or EV (white bars).

The impact of external Cs^+^ on cell growth depended not only on Kup but also on other K^+^ uptake systems (TrkG, TrkH and Kdp) as shown in Figs 1 and 2. To evaluate the contribution of these other K^+^ uptake transporters to adaptation to Cs^+^ stress, Kup was expressed in *E*. *coli* LB2003 which is lacking activity of four K^+^ transporters, Kup, Kdp, TrkG and TrkH^23^ (Fig. 3). The growth of both LB2003 harboring the empty vector (EV) and LB2003 expressing Kup increased with the increase in K^+^ concentration in the medium. The growth of LB2003 containing Kup decreased with higher concentrations of Cs^+^ in the medium, whereas the control strain containing EV was much less sensitive to Cs^+^ and even showed slightly increased growth at higher Cs^+^. Remarkably, high Cs^+^ concentrations, e.g. 20 mM Cs^+^ in the absence or presence of 1, 5, 10 or 20 mM K^+^ severely impaired growth of LB2003 expressing Kup compared to the control cells containing EV (Fig. 3e). Kup was therefore responsible for the hypersensitivity of the cells to excess Cs^+^. The growth of LB2003 expressing Kup was significantly lower compared with WT *E*. *coli* under the same conditions (e.g. 1 mM K^+^ and 0, 1, 10 mM Cs^+^, Figs 2b and 3b). The positive effect of Cs^+^ on WT cell growth (at 25 mM Cs^+^, Fig. 1a) largely disappeared in the K^+^ transporter mutant LB2003 (at 20 mM Cs^+^, Fig. 3a). This indicated that other K^+^ uptake systems (Trk and Kdp) besides Kup were important to sustain the growth of the wild-type cells during high Cs^+^ stress.

### Role of Kup in uptake of Cs^+^ into the cells and export of K^+^

**Figure 4:**
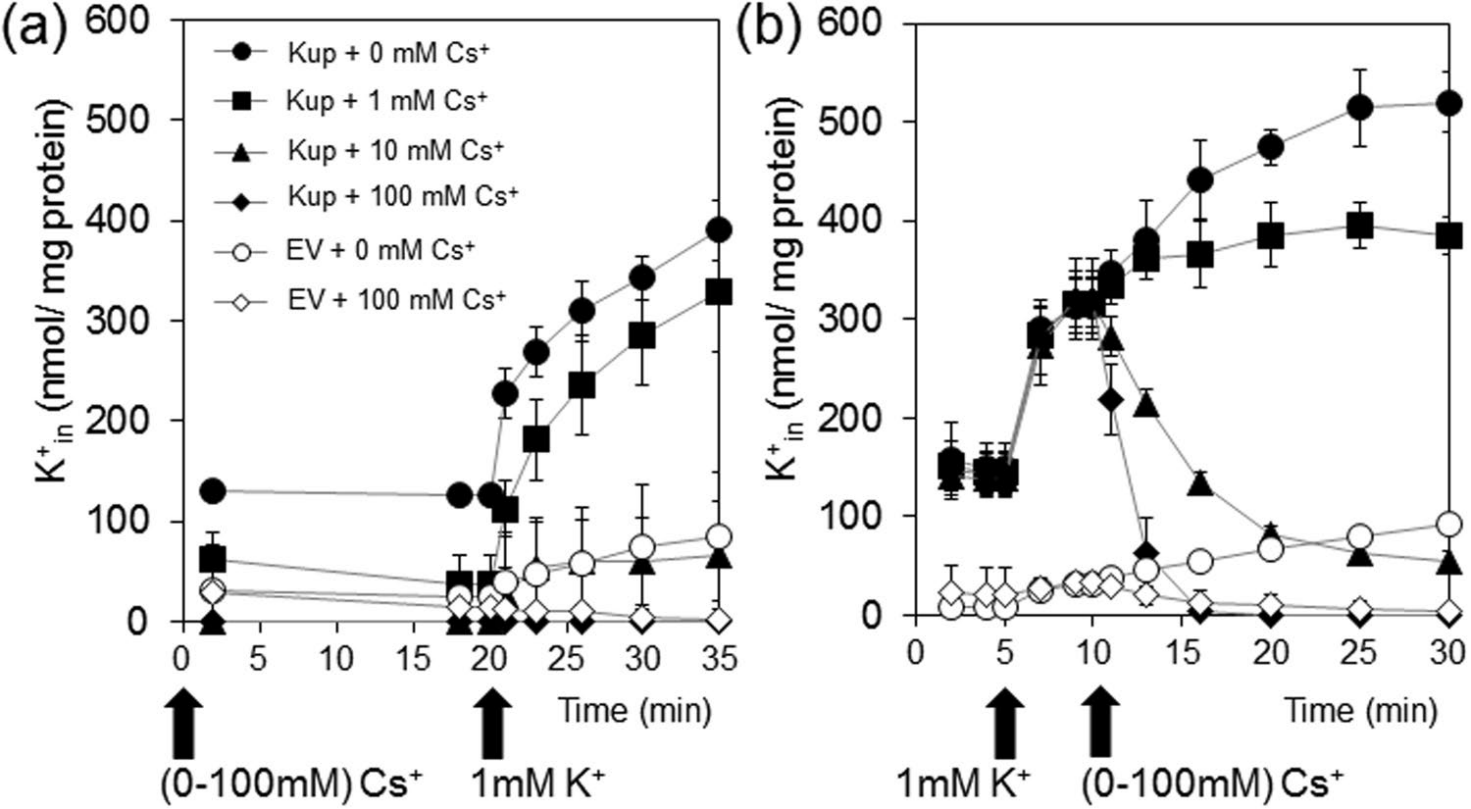
High concentrations of Cs^+^ reduced K^+^ content in cells expressing Kup. Cs^+^ (0, 1, 10 or 100 mM) was added 20 min before (**a**) or 5 min after (**b**) K^+^ addition (1 mM final concentration). The error bars represent mean ± S.D. (n = 3). Filled and open symbols represent *E*. *coli* LB2003 transformed with Kup and EV, respectively.

**Figure 5:**
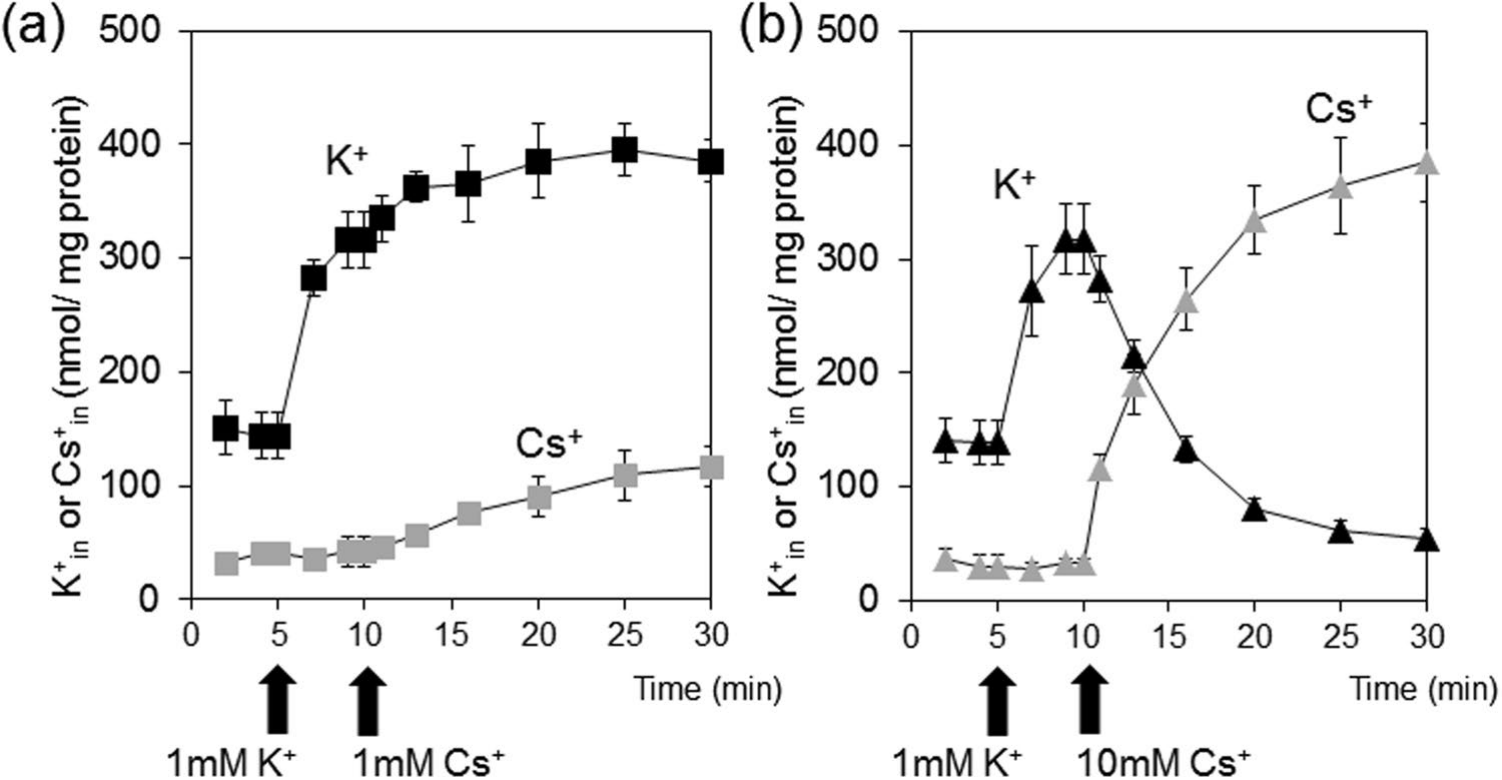
Loss of intracellular K^+^ due to Kup-driven Cs^+^ influx. The Cs^+^ content (gray symbols) was determined from the same *E*. *coli* LB2003 expressing Kup cells shown in Fig. 4b. Final concentration of Cs^+^ added in the medium was 1 mM (**a**) or 10 mM (**b**). The data representing K^+^ content (black symbols) is shown here for comparison, it is the same as that in Fig. 4b. The error bars represent mean ± S.D. (n = 3).

To dissect the effect of Cs^+^ on Kup-mediated K^+^ uptake, K^+^ content in LB2003 expressing Kup was measured in the buffer supplemented with different amounts of K^+^ and Cs^+^. The cells that were pretreated without or with 1 mM Cs^+^ showed a rapid increase in intracellular K^+^ content after K^+^ addition (Fig. 4a). In contrast, pretreatment with 10 or 100 mM Cs^+^ only led to a very slow increase of intracellular K^+^ content. The antagonistic effect of Cs^+^ influx on intracellular K^+^ content was more pronounced when Cs^+^ was added to LB2003 expressing Kup that had been pretreated with buffer containing 1 mM K^+^ (Fig. 4b). This effect was concentration-dependent, when low concentrations of Cs^+^ (0 or 1 mM) were added, the cellular K^+^ content continued to increase, whereas higher concentrations of Cs^+^ (10 or 100 mM) coincided with a loss of intracellular K^+^ (Fig. 4b). These results indicate that high Cs^+^ in the medium led to removal of K^+^ from the cells. This was confirmed by measurement of the intracellular concentrations of Cs^+^ of the cells treated with 1 mM K^+^ prior to addition of 1 mM or 100 mM Cs^+^ shown in Fig. 4b (Fig. 5). When 1 mM Cs^+^ was added to the cells the K^+^ content remained higher than that of Cs^+^, indicating that Kup transported K^+^ at a higher rate than Cs^+^ into the cells. Kup therefore recognized K^+^ as a preferential cation compared to Cs^+^ (Fig. 5a). Higher medium concentrations of Cs^+^ (10 mM) resulted in much higher Cs^+^ content compared with 1 mM Cs^+^ and a strong decrease in K^+^ content, suggesting that accumulated K^+^ was extruded from the cells (Fig. 5b).

### Requirement of Kdp to *E*. *coli* under high external Cs^+^ concentrations

**Figure 6:**
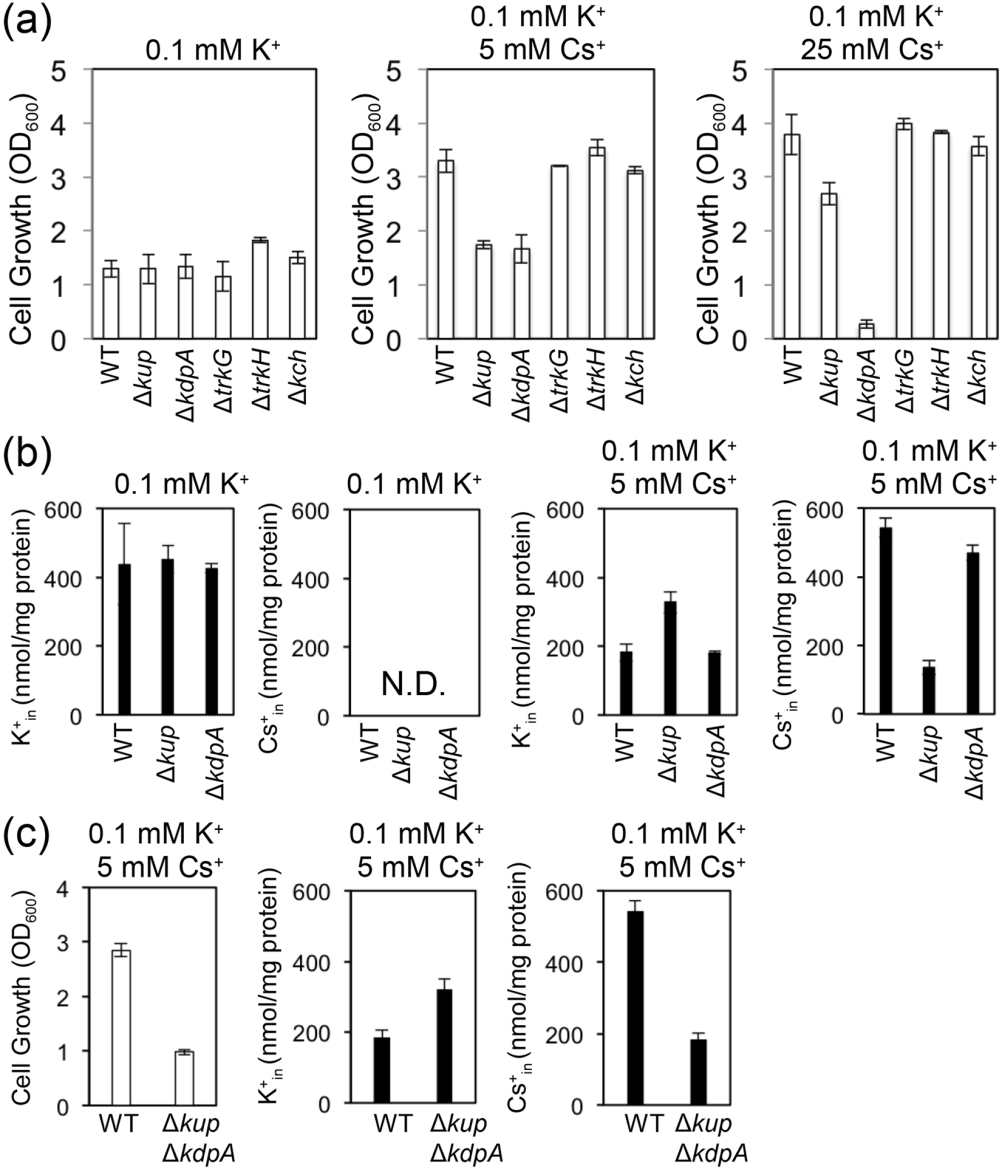
Requirement of K^+^ uptake transporters under high Cs^+^ conditions. (**a**) Growth assay of *E*. *coli* WT, Δ*kup*, Δ*kdpA*, Δ*trkG*, Δ*trkH* and Δ*kch* in minimal medium containing 0.1 mM K^+^ without or with 5 mM or 25 mM Cs^+^, which was performed in the same experimental condition to that of Fig. 1. (**b**) The content of K^+^ and Cs^+^ of WT, Δ*kup* and Δ*kdpA* grown in minimal medium containing 0.1 mM K^+^ with 5 mM Cs^+^ measured from the data in Fig. 6a. N.D. Not detected. (**c**) The growth of WT and Δ*kup*Δ*kdpA* and their content of K^+^ and Cs^+^.

The results above indicate that Kup conferred Cs^+^ tolerance to the cells by cooperating with other K^+^ uptake transporters in maintaining the intracellular K^+^ level. To further explore the importance of these other K^+^ uptake transporters in the presence of high Cs^+^, we performed growth tests of *E*. *coli* mutants carrying single deletions in the genes encoding TrkG, TrkH, Kdp or Kch in medium containing 0.1 mM K^+^ and varying concentrations of Cs^+^. We included Δ*kch* in this experiment because *E*. *coli* also contains a K^+^ channel homolog, Kch, whose function is unknown^24, 25^. At 5 mM Cs^+^, the growth of Δ*kdp* was similar to Δ*kup* but lower than that of WT, Δ*trk*G, Δ*trk*H and Δ*kch* (Fig. 6a). Higher concentrations of Cs^+^ (25 mM) severely impaired the growth of Δ*kdp*. This suggested that under high Cs^+^ and low K^+^ conditions, Kdp contributed to the uptake of K^+^ into the cells while Kup took up Cs^+^. As expected, the content of K^+^ in Δ*kdp* was lower than that in Δ*kup* (Fig. 6b). Due to the lack of growth of Δ*kdp* at 25 mM Cs^+^, the content of K^+^ and Cs^+^ was not measured under those conditions. Kdp is a high affinity K^+^ pump in *E*. *coli* ^26^. To further evaluate the connections between Kdp-mediated K^+^ uptake and Kup-mediated Cs^+^ uptake, the double knockout mutant Δ*kdp*Δ*kup* was generated (Fig. 6c). The double knockout mutant grew less well than either single mutant under the same conditions (5 mM Cs^+^ and 0.1 mM K^+^). The content of K^+^ and Cs^+^ was comparable to that of Δ*kup*. This implies that the reloading of K^+^ into Δ*kdp*Δ*kup* might occur through Trk (TrkG and TrkH) and/or another unidentified K^+^ transport system. These results revealed that Kdp for K^+^ uptake contributes to sustain cell growth by Kup-mediated Cs^+^ uptake under excess Cs^+^ and limiting K^+^ conditions.

## Discussion

**Figure 7:**
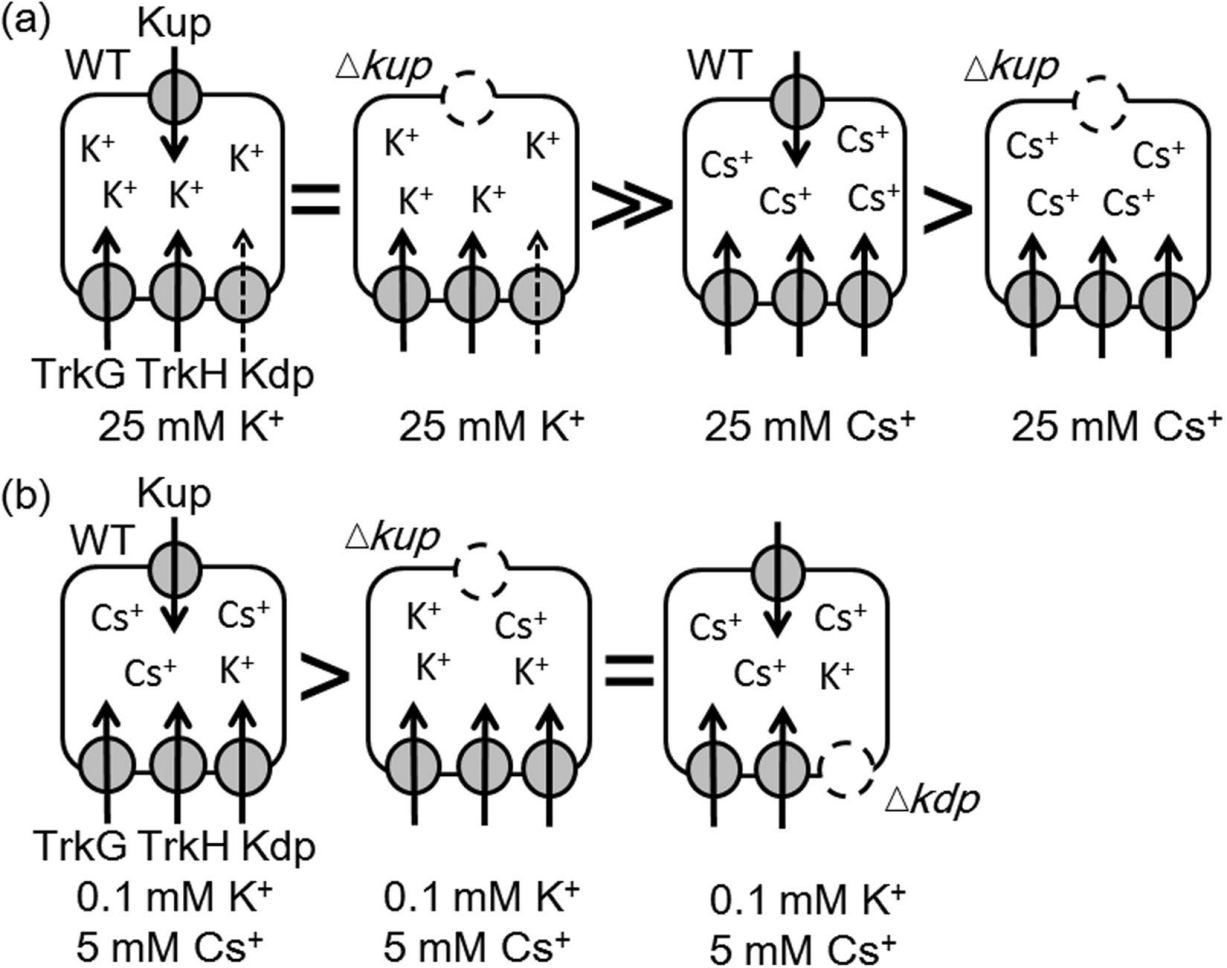
Model explaining the coordinated activity of Kup with the K^+^ uptake transporters Kdp and Trk in response to excess Cs^+^ in *E*. *coli*. (**a**) Kup-mediated Cs^+^ uptake contributed to growth of *E*. *coli* WT in medium containing Cs^+^ as the only alkaline metal. Under sufficient K^+^ conditions, loss of Kup had no effect on growth. Contribution of high affinity Kdp uptake system may be small at high K^+^ and their expression may be suppressed at high K^+^^31, 38^. (**b**) As long as a low concentration of K^+^ was present, the WT was able to grow even with high amounts of Cs^+^. (**c**) K^+^ uptake by other K^+^ uptake transporters (Kdp, TrkG and TrkH) was important for the growth of *E*. *coli* in the presence of high Cs^+^. = , >>, > indicates equal, much higher and higher growth.

This study showed that Cs^+^ uptake by Kup promoted cell growth under K^+^-limiting conditions. The positive effect of Cs^+^ on *E*. *coli* growth was reduced when the amount of K^+^ in the medium was increased at the same time. Other K^+^ uptake systems, TrkG, TrkH and Kdp likely also contributed to cell tolerance to high Cs^+^. Specifically, Kdp helped to prevent depletion of intracellular K^+^ due excess Cs^+^ (Fig. 7). The activities of Kup and Kdp were coordinated to support cell growth during excess Cs^+^ conditions in *E*. *coli*.

Several studies have found that other cations like Na^+^, Rb^+^ or Cs^+^ can substitute for intracellular K^+^ under K^+^-limited conditions. Rice accumulates Na^+^ to compensate for K^+^ starvation^27^, olive trees (*Olea europaea* L.) are able to replace K^+^ with Na^+^^28^, while pint bean subtitutes by Rb^+^ and Na^+^ for K^+^ limitation^22^, and turtle heart cells are capable of replacing K^+^ with Cs^+^^29^. However, an adverse effect of Cs^+^ accumulation has also been reported. In cyanobacterium *Synechocystis* PCC 6803, addition of 1 mM Cs^+^ increases the doubling time by 64%, compared to control medium^9^. Similarly, in the green algae *Chlorella emersonii* addition of 1 mM Cs^+^ into the medium increases the doubling time by 34%^10^. In *E*. *coli* and *Bacillus subtilis*, Cs^+^ toxicity is found to be dependent on external pH and the presence of other cations, especially K^+^ and Na^+^. Based on an agar diffusion assay, *E*. *coli* has a minimum inhibitory K^+^: Cs^+^ concentration ratio of 1: 6^30^. Cs^+^ is therefore likely a partial substitute alkaline ion for K^+^, but as shown by this study, it does not fully compensate for K^+^.

What is the cause of Cs^+^ toxicity for the cells? Cs^+^ is known to compete with K^+^ binding sites on K^+^ channels and K^+^ dependent enzymes and thereby to inhibit their function. Hampton *et al*. emphasized that the toxicity of Cs^+^ is mainly caused by competition between K^+^ and Cs^+^ for K^+^ association sites on vital proteins, not by blockage of K^+^ uptake resulting in K^+^ starvation alone^8^. A further aspect of Cs^+^ cytotoxicity is that Cs^+^ interferes with protein expression^8^. The gene expression of K^+^-repleted cells differs from that of K^+^-depleted cells due to intracellular accumulation of Cs^+^. This implies that prolonged Cs^+^ accumulation in the cells alters the cellular response. To rescue the detrimental effect of Cs^+^ on cells under high Cs^+^ conditions, K^+^ uptake transporters likely function in replenishment of K^+^ when the K^+^ concentration in the medium is low^30^. Figure 3 shows that at 25 mM Cs^+^ in K^+^-limited medium (0.1 mM K^+^), the K^+^ inside of the cells was kept at a low level (152 nmol K^+^/mg of protein). Despite of this, the cells still grew well (Figs 2a and 7). The basal level of K^+^ in the cells likely alleviated the effect of the intracellular over-accumulation of Cs^+^. This explains well why K^+^ depletion in the presence of Cs^+^ severely hampered cell growth (Fig. 1a). Kup apparently facilitates the uptake of Cs^+^ as an electroneutral substitute for K^+^ as an osmo-protectants, and Cs^+^ does not act as a substitute for other physiological roles of K^+^^10^.

An alternative role of Kup under conditions of high external Cs^+^ may be the induction of the high affinity K^+^-uptake Kdp system, which takes up K^+^ into the cells at low K^+^. The *K*_m_ value for K^+^ of Kdp is much lower than that of Trk, and both Kdp and Trk can distinguish K^+^ from Cs^+^^11^. In *E*. *coli*, Kdp is expressed upon K^+^ depletion^26^. The *kdp* operon is also induced in cells with low internal K^+^ due to intracellularly accumulated Cs^+^^31^. A relative increase of Cs^+^ accumulation induced by K^+^ loss was also reported in Chlorella^10^. K^+^ pumping by Kdp might be induced to supply K^+^ from low K^+^ in the medium because Kup-mediated Cs^+^ accumulation results in a lower intracellular K^+^/Cs^+^ ratio (Fig. 6). When Kup-mediated Cs^+^ uptake promoted *E*. *coli* growth in medium containing low amounts of K^+^, Kdp may enable the cells to maintain the proper intracellular balance of K^+^/Cs^+^ to protect themselves from Cs^+^ toxicity^8^.

The *K*_m_ value of Kup for K^+^ and Cs^+^ was reported to be approximately 0.37 mM and 5 mM, respectively^11^, indicating that Kup preferred to transport K^+^ over Cs^+^. This property of Kup was also seen in our experiments (Figs 2 and 5a). The rate of K^+^ uptake by Kup was faster than that of Cs^+^ (Fig. 5a). Figure 2b also showed that lack of Kup resulted in a decrease of 8.8–25% in K^+^ accumulation in cells treated with varying concentrations of Cs^+^. Moreover, in the presence of 1 mM K^+^ in the medium there was no significant difference in Cs^+^ content in WT and Δ*kup* (Fig. 2b), indicating that under sufficient K^+^ conditions Kup-mediated Cs^+^ uptake activity was low.

The remaining Cs^+^ influx activity in Δ*kup* shown in Fig. 2b was not due to Kup function. Considering that Trk (TrkG and TrkH) and Kdp have only negligible Cs^+^ uptake activity^11^, *E*. *coli* likely possesses another, yet unidentified Cs^+^ uptake transport system in addition to Kup.

Another insight obtained in this study is that K^+^ efflux from K^+^-loaded cells was caused by Cs^+^ accumulation in the cells (Fig. 5). *E*. *coli* contains a glutathione-gated KefB/KefC system^32, 33^ and a K^+^/H^+^ antiporter^34^ in the inner membrane. It remains to be shown whether Kup provides a K^+^ efflux pathway across the inner membrane or whether other transporters mediate K^+^ efflux.

In summary, Kup took up Cs^+^ to support *E*. *coli* growth in K^+^-depleted medium. However, the growth rate of cells under Cs^+^ stress conditions depended on the activity of K^+^ uptake transporters. The temporary increase of intracellular Cs^+^ driven by the activity of Kup might in turn stimulate another K^+^-selective uptake system, Kdp to increase the cellular K^+^ content, which partially offset the effect of accumulated Cs^+^ in the cells. The relative broad selectivity of Kup, which is not found in Trk and Kdp, may contribute to increased plasticity in the cellular response to non-physiological levels of alkali metals in the environment.

## Methods

### Growth test condition

*E*. *coli* strain BW25113 (wild type, WT), single knockout mutants of *kup* (Δ*kup*), *kdp* (Δ*kdp*), *trkG* (Δ*trkG*) and *trkH* (Δ*trkH*) in the BW25113 background were provided by the National BioResource Project (NIG, Japan)^35^. The double mutant of Δ*kup*Δ*kdp* was generated by PCR-based mutagenesis^36^. Briefly, the *kup* gene was deleted in the Δ*kdp* strain using PCR products amplified with two primers, 5′-AAGCACACATTTCATATTTCAACGAAAGACTAGTCTATGATTCCGGGGA TCCGTCGAC-3′ and 5-GAAAGGAGGCGTCTGGCGTTAGATTTCGACCTGAGTACCTG TAGGCTGGAGCTGCTTC-3. The kanamycin cassette was removed from the resultant *E*. *coli* mutant^36^. All strains were grown in medium containing 0.5% yeast extract, 0.5% KCl, and 1% polypeptone. Pre-cultures were grown in liquid minimal medium (46 mM Na_2_HPO_4_, 23 mM NaH_2_PO_4_, 8 mM (NH_4_)_2_SO_4_, 0.4 mM MgSO_4_, 6 mM FeSO_4_, 10 μg/ml thiamine and 1% glucose) supplemented with 1 mM KCl^16^. The pre-cultures were incubated at 30 °C for 24 h. Cells were harvested by centrifugation (15,000 rpm for 1 minute), washed with K^+^-free buffer (46 mM Na_2_HPO_4_, 23 mM NaH_2_PO_4_, 8 mM (NH_4_)_2_SO_4_) then re-suspended with the same buffer. The cell suspension was inoculated (1%) into the same liquid minimal medium containing various concentrations of KCl, RbCl and CsCl. The cultures were incubated at 30 °C for 24 h. The growth of *E*. *coli* WT and Δ*kup* was determined by measuring the optical density at 600 nm (OD_600_). The cation content of the cell pellets was determined using an atomic absorption spectrometer (iCE 3500 Thermo Fisher Scientific AA Spectrometer).

### Measurement of K^+^ uptake in *E*. *coli*

*E*. *coli* strain LB2003 (F^−^, *thi*, *lacZ*, *gal*, *rha*, Δ*kdpFABC5*, *trkD1*, Δ*trkA*), which lacks activity of the three K^+^ uptake systems, was transformed with pPAB404 empty vector (EV) or pPAB404-EcKup-6xHis (Kup) as described previously^16^. The cation uptake procedure was essentially conducted as described previously with some modifications^37^. Briefly, *E*. *coli* LB2003 containing EV, Kup or Kdp were cultured in minimal medium supplemented with 30 μg/ml ampicillin and 20 mM KCl at 30 °C for 24 h. Pre-cultures were inoculated into the minimal medium to which 0.1 mM isopropyl *β*-D-1-thiogalactopryanoside (IPTG) was added and then incubated at 30 °C. Overnight-grown cultures were harvested by centrifugation. The cells were re-suspended in 120 mM Tris-HCl (pH 8.0) and 1 mM EDTA, incubated 30 min at 37 °C, collected by centrifugation and washed three times with 50 mM Tris-HCl pH 7.5, then re-suspended with the same buffer. After shaking at room temperature for 20 min, the concentration of the cell suspensions was adjusted to an OD_578_ of 3.0 with the same buffer. For the experiments where Cs^+^ was added prior to K^+^ supplementation, 10 mM of glucose and various concentrations (0, 1, 10, or 100 mM) of CsCl were added to the cells 20 minutes before K^+^ addition (t = 0 min). At t = 20 min, KCl was added at a final concentration of 1 mM. For the experiments where K^+^ was added prior to Cs^+^ supplementation, 10 mM of glucose was added at t = 0 min, then 1 mM KCl was added at t = 5 min and various concentrations (0, 1, 10, or 100 mM) of CsCl were added into the cells at t = 10 min. For sampling, aliquots of 1 mL were taken at different times (t = 1, 3, 6, 10, 15, and 20 min), and centrifuged for 1 min through silicone oil (Sigma Aldrich). The supernatant was removed by using an aspirator, then the pellet was disrupted by addition of 5% trichloroacetic acid and heating at 100 °C for 5 min. The total protein content in the cell pellets was measured by using the BCA protein assay kit (Micro BCA Protein Assay, Thermo Scientific). The K^+^ content of the cell pellets was determined using an atomic absorption spectrometer (iCE 3500 Thermo Fisher Scientific AA Spectrometer).
